# Reconstruction of productivity signal and deep-water conditions in Moroccan Atlantic margin (~35°N) from the last glacial to the Holocene

**DOI:** 10.1186/s40064-015-0853-6

**Published:** 2015-02-10

**Authors:** Yassine El Frihmat, Dierk Hebbeln, EL Bachir Jaaidi, Nadia Mhammdi

**Affiliations:** Faculty of Sciences, Mohammed V University, Agdal Rabat, Morocco; Center for Marine Environmental Sciences (MARUM), University of Bremen, Leobener Str., 28359 Bremen, Germany; Scientific Institute, Mohammed V University, Agdal, Rabat, Morocco

**Keywords:** Paleoproductivity, Larache margin, Late quaternary, Total organic carbon, Carbonate, Planktonic δ^13^C

## Abstract

In order to assess the changes in sea-surface hydrology and productivity signal from the last glacial to the Holocene; a set of isotopic, geochemical and microgranulometric proxies was used for this study. Former studies revealed that the reconstruction of paleoproductivity from ocean sediment gives different results depending the measurement used. The comparison between our productivity proxies (total organic carbon, carbonate and planktonic δ^13^C) as well as previous results in nearby location indicates that the planktonic δ^13^C responds better to marine productivity changes and represents therefore a suitable proxy for paleoproductivity reconstruction in our studied area. The productivity signal reveals two main enrichments during the Young Dryas (YD) and the Heinrich Event 1 (HE 1) and correlates perfectly with upwelling activity mentioned by an increasing trend of aeolian proxies. In addition, our results show that biogenic components in the sediment have a marine origin and the proportion of organic matter preserved depends on the total sediment accumulation rate.

## Introduction

The abundance and distribution of biogenic particles in the surface waters depend on the amount of nutrients supplies of fluvial input or by the amount of nutrient-rich water upwelled. To find out how the efficiency of the biogenic production has changed from a cold to warm climatic stage in Moroccan Atlantic margin (~ 35°N), it is important to evaluate the modification of the productivity pattern.

Deep-sea sediments off Northwest Africa have been studied by many authors in order to obtain information concerning the Paleoceanography of the Northeast Atlantic and climatic evolution of the African continent (e.g., Parkin and Shackleton 1973; Pastouret et al. 1978; Koopmann 1981; Sarnthein et al. 1982; Diester-Haas 1983; Ganssen and Sarnthein 1983; Thiede 1983; Stein and Sarnthein 1984; Jaaidi and Cirac 1987; Jaaidi 1993; Sánchez-Goñi et al. 2002; Cacho et al. 1999; Moreno et al. 2002; Martrat et al. 2004; Eberwein and Mackensen 2008; Penaud et al. 2010; De Jonge 2010; Wienberg et al. 2010…). The productivity conception has changed over time; at first generally increased glacial productivity was proposed for the entire NW-African margin (e.g. Sarnthein et al. 1988). Gradually, this uniformity concept was ignored, in fact, enhanced glacial productivity was observed at 25°N (Bertrand et al. 1996; Abrantes 2000; Sicre et al. 2000; Ternois et al. 2000). On the other hand, glacial productivity was lower off Cape Blanc (21°N) (Zhao et al. 2000 Sicre et al. 2001; Henderiks and Bollmann 2004). This underlines that strong productivity differences co-exist within regionally small areas (Bertrand et al. 1996). Furthermore, the reconstruction of paleoproductivity from ocean sediment gives different results depending on the measurement used (Lazarus et al. 2006).

**Figure 1:**
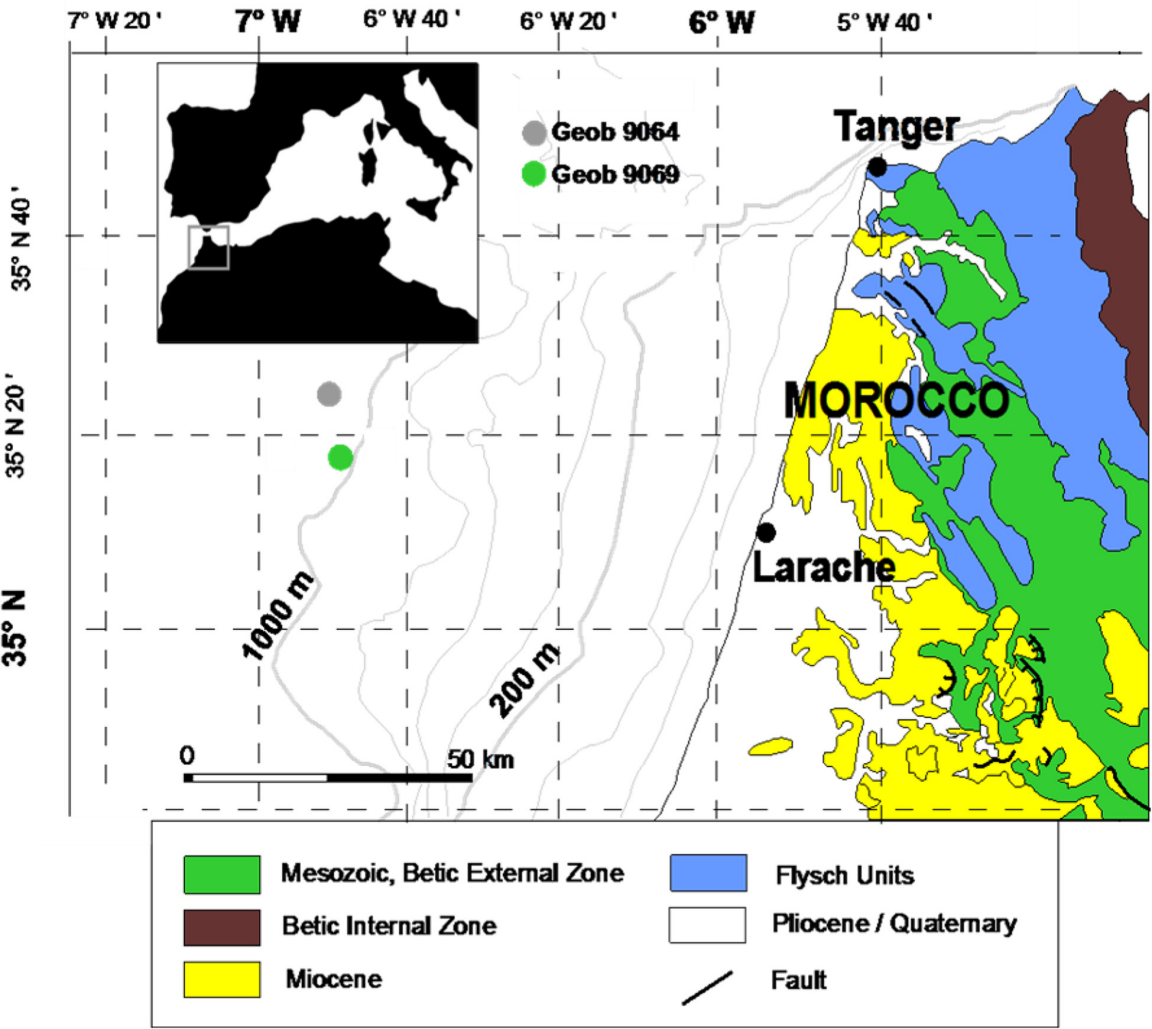
**Geological map of the Gulf of Cádiz (from** **Medialdea et al.** 2009 **) showing the cores distribution in the studied area.**

In this regard and in order to determine which parameter able to reflect better the paleoproductivity changes, we used a multi-proxy approach based on planktonic δ^13^C, organic carbon and carbonate. In addition, we will focus on the detailed reconstruction of the paleoproductivity pattern at Moroccan Atlantic margin (~35°N) (Figure 1), and follow their variations through the time from the last glaciation to the Holocene. Finally, we will point out the potential causes for cyclic paleoproductivity variations and the probable factors influencing organic carbon preservation.

## Methodology

While coastal upwelling occurs mostly on the shelf, biogenic particles derived from upwelling are deposited mostly at upper continental slope due to remobilization and transport across the shelf (Fütterer 1983). Therefore, to describe the Glacial−Holocene changes of paleoproductivity, two sediment cores (Figure 1, Table 1) located at the continental slope are investigated in this study. They were recovered during Pelagia Cruise 64PE284 in February 2008.

**Table 1:** **Positions of the sediment cores investigated for this study**

**Cores**	**Latitude °N**	**Longitude °W**	**Water depth (m)**	**Recovery (m)**
GeoB 9064	35:24.91	6:50.71	702	5.44
GeoB 9069	35:18.21	6:49.14	669	5.13

CHN analysis (Total Organic Carbon (TOC), Carbonate and C/N ratio) have been carried out in the department of Geosciences in Bremen University. Sediment samples taken from each 5 cm were freeze-dried and homogenized. Two precise amounts (25 mg) of sediment are taken for each sample of which one Inorganic Carbon (IC) was removed by addition of 1 N HCl. Total carbon (TC) and total nitrogen (TN) concentrations were measured on non-acidified samples, while Organic Carbon (TOC) was measured on acidified samples using a CHN-Analyzer (Haereus).

Carbonate content is another important aspect in relation to marine productivity and carbonate preservation/dissolution. The bulk carbonate content (% weight CaCO_3_) was calculated assuming that calcium carbonate was the only carbonate-bearing mineral:

$$ \mathrm{C}\mathrm{a}\mathrm{C}{\mathrm{O}}_3=\left(\mathrm{T}\mathrm{C}\hbox{--} \mathrm{T}\mathrm{O}\mathrm{C}\right)*8.333. $$

Geochemical analysis are done by XRF Core Scanner (X-Ray Fluorescence) which is an instrument designed and manufactured in the Netherlands at the Netherlands Institute of Sea Research (NIOZ). It is capable to give in 100 minutes a chemical analysis from Aluminum to Iron along one meter section of a sediment core with a sampling resolution of 1 cm. This non-destructive analysis gives the results for each analyzed element in CPS (counts per second). The analyses are carried out directly on the surface of the sediment cores, no sampling or preparation is necessary. In this study the ration Fe/Ca will be used for correlation between cores and Fe intensity will be used as a proxy for aeolian terrigenous input.

The δ^18^O and the δ^13^C isotopic signals from planktonic (*Globigerinoides ruber*) and benthic foraminifera (*Cibicides wuellerstorfii*) were measured in order to establish a reconstruction of plaeoclimate and deep water circulation. Sometimes, *C.wuellerstorfi* is almost absent, hence we used *Uvigerina peregrina* as it calcifies its test close to equilibrium of the bottom water δ^18^O (Shackleton 1974; McCorkle et al. 1990). On average, five to seven individuals of foraminifera were handpicked from the > 150 μm size fraction of each sample, sufficient to reach the minimum weight of material (180 μg) detectable by the mass spectrometer. Oxygen and carbon isotopic data obtained are reported in the usual notation, which is referred to the PeeDee belemnite (V-PDB) standard. The benthic isotope were measured in the Department of Geosciences (FB5-Geowissenschaften) at Bremen University using a Finnigan MAT 252 mass spectrometer with a precision of ± 0.07‰ for δ^18^O and ± 0.05‰ for δ^13^C.

## Results

### Age model

**Figure 2:**
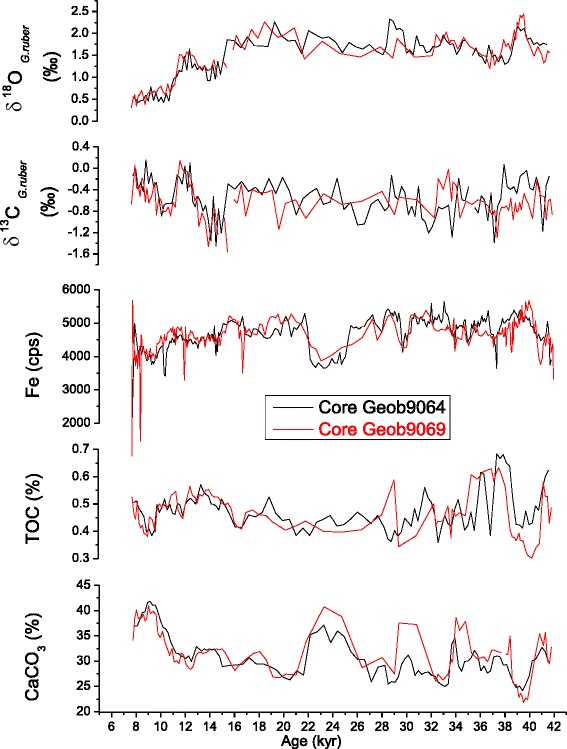
**Correlation of the gravity cores using the isotopic data (planktonic δ** ^**13**^ **C and δ** ^**18**^ **O), the organic carbon (TOC), the carbonate (CaCO**_**3**_ **) and the Fe content.** Geob 9064 was chosen as reference.

Age Model of the core Geob 9069 was obtained by correlating the isotopic data, the organic carbon, the carbonate and the Fe content (Figure 2) with the core Geob 9064 which has been dated by ^14^C. Ages between the tie points were obtained by linear interpolation. The major transition Holocene-Last Glaciation could be easily identified in all cores.

### Oxygen isotope

Significant changes in planktonic δ^18^O during the last ~ 30 Kyr are most likely caused by monsoon-induced salinity fluctuation (Duplessy 1982; Kudrass et al. 2001) and suggest large changes in monsoonal precipitation. Variations in isotopic data enable then the reconstruction of past changes in paleomonsoon intensity.

In our study, the δ^18^O values (Figure 2) vary in a similar pattern in the two cores and appear to track one another. The prominent low δ^18^O values during the Holocene suggest increased sea surface temperature and decreased salinity which highlight the influx of freshwater as a result of intensified monsoonal precipitation. In contrast, during the glacial period, increasing of planktonic δ^18^O values shows that the solar insolation was stronger. On shorter timescale, δ^18^O record exhibits high amplitude fluctuations indicating seasonal variations of monsoon precipitation. A prominent feature of δ^18^O variations is a clear increase at ~ 11 kyr and 16 kyr, what could give an accurate pinpointing of the Younger Dryas (YD) and the Heinrich Event 1 (HE 1).

### Carbon isotope

The planktonic δ^13^C is usually used as a paleoproductivity proxy in surface waters (Berger et al. 1978). The comparison between the planktonic isotopic values reveals perfect correlation between the δ^18^O and the δ^13^C records (Figure 2). During the late Holocene, the planktonic δ^13^C values exhibit a decreasing trend, the YD and the HE 1 show two main enrichments and the last glacial is marked generally by heavier values.

### Iron (Fe)

In order to determine how productivity would depend on wind strength, we used the Fe intensity record as an indicator for the long-term trends of terrigenous input and assume that higher Fe content in the sediment record reflects periods of enhanced dust input (Rogerson et al. 2006; Mertens 2009).

The Fe content and the planktonic δ^13^C show approximately similar profiles (Figure 2), low values were recorded until around 10 kyr, while the YD and the HE 1 display noticeable increasing. The last glacial reveals relatively high Fe values, however we denote a clear dip during the LGM until the onset of the HE 2.

### Organic carbon and carbonate

In oligotrophic areas situated well above lysocline, carbonate accumulation may serve as an indicator of primary productivity. In contrast, in upwelling areas, organic carbon accumulation may be better (Rühlemann et al. 1996).

The Total organic carbon (TOC) and the Carbonate profiles exhibit differential variations (Figure 2); equally, we denote the absence of a clear correlation with the parameters presented above which makes hard to choose the adequate proxy to decipher the variation of the paleoproductivity in our studied area.

## Discussion

Coastal upwelling regions are some of the most productive regions in the world’s ocean; some works have demonstrated that changes in atmospheric circulation had consequences on the dynamic of upwelling systems basically controlled by the activity of the trade-winds (Pearce 1991; Hagen 2001; Mann and Lazier 2005; McGregor et al. 2007). In this study we set out to reconstruct the productivity variations in Moroccan Atlantic margin (~35°N) since the last glacial, equally, we aim to determine the potential causes of the paleoproductivity variations.

**Figure 3:**
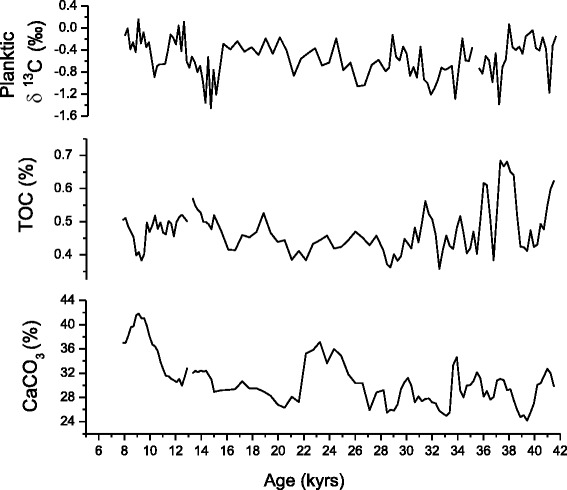
**Comparison versus ages of the productivity proxies (Geob 9064).**

The reconstruction of paleoproductivity from ocean sediment gives different results depending on the measurement used (Lazarus et al. 2006). A quick overview on (Figure 3) exhibits a clear difference between the parameters of the same core (Geob 9064) which makes hard to extract a common interpretation of paleoproductivity, whence the necessity to choose carefully the parameter that represents better the paleoproductivity.

In this regard, and in order to determine which parameter able to reflect better the paleoproductivity changes, we will follow the variation of our productivity proxies (TOC, carbonate and planktonic δ^13^C) to determine the parameter which correlate better with the variation of wind strength and hence the upwelling activity which can give idea about the paleoproductivity. In addition, we will discuss the origin of the organic carbon preserved in marine sediments and determine the factors controlling its burial.

### Paleoproductivity

#### Total organic carbon

High primary production causing a great flow of organic matter down to the sea floor supports an increased preservation of organic carbon in the sediment. Hence, the observation that the distribution of organic carbon contents in marine sediments matches the pattern of primary production (e.g. Sarnthein et al. 1988; Lyle et al. 1988; Berger and Herguera 1992; Freudenthal et al. 2002; Jahn et al. 2003) is the basis for using organic carbon as an indicator of paleoproductivity.

In our results, this assumption is supported by the mismatch between TOC maxima and peaks in the Fe record (i.e. Geob 9064, Figure 2); we therefore suggest that variations in TOC may be attributed to changes in marine productivity and not to fluctuation in terrigenous inputs (Jahn et al. 2003).

**Figure 4:**
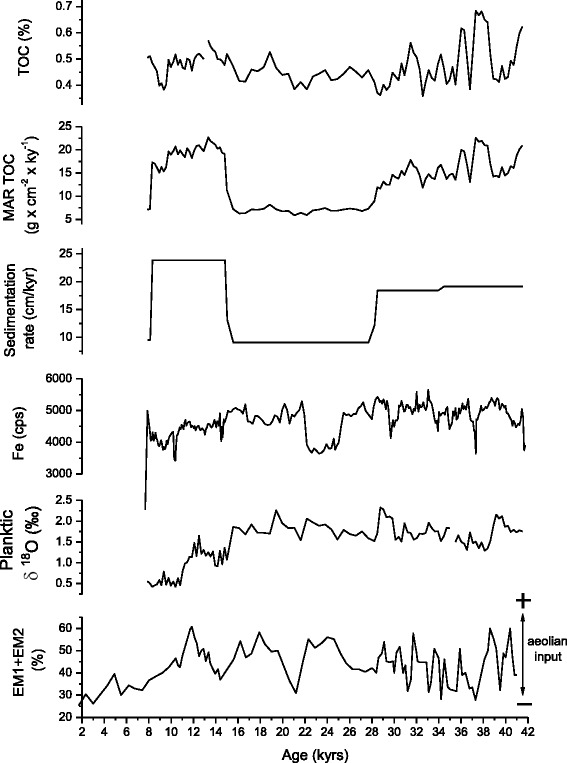
**Comparison of the total organic carbon (TOC) and the TOC mass accumulation rates (TOCMAR) of Geob 9064 with the sedimentation rate pattern, the planktonic δ** ^**18**^ **O and the terrigenous inputs (Fe).** The aeolian input record (EM1+EM2) refers to Wienberg et al. (2010).

The comparison of the total organic carbon (TOC) and TOC mass accumulation rates (TOCMAR) of Geob 9064 displays noticeable differences especially seen between 14 kyr and 28 kyr when TOC displays significant fluctuations while TOCMAR shows a maintaining of low values correlating with the sedimentation rate pattern (Figure 4). We therefore deduce that variations in carbon organic may be attributed to changes in either productivity or preservation by increased sedimentation rates or both (Emerson et al. 1985; Emerson and Hedges 1988; Hedges and Keil 1995).

The problem is that there is no clear relationship between marine productivity and sedimentation rates, we can have low productivity but we can have differences in terrigenous input giving quite different organic carbon content if we transfer to accumulation rates. For example 7 kyrs and 19 kyrs show respectively drop and peak in TOC leading us to predict low and high productivity were expressed in these times; in contrast, TOCMAR reveals low values indicating the inverse.

Consequently, the high organic carbon content shown in interglacial times is just an artifact of better preservation of organic matter due to high sedimentation rate and not only to variations in marine productivity.

Furthermore, enrichment in TOC and TOCMAR do not coincide with maxima of planktonic δ^18^O values and aeolian input record calculated by Wienberg et al. (2010) which points to periods of intensified trade wind and resulting changes in upwelling intensity (Figure 4).

Thus as a proxy for productivity, the organic carbon content is unhelpful, while the TOCMAR can be used to decipher the deposition conditions of organic matter.

#### Carbonate

**Figure 5:**
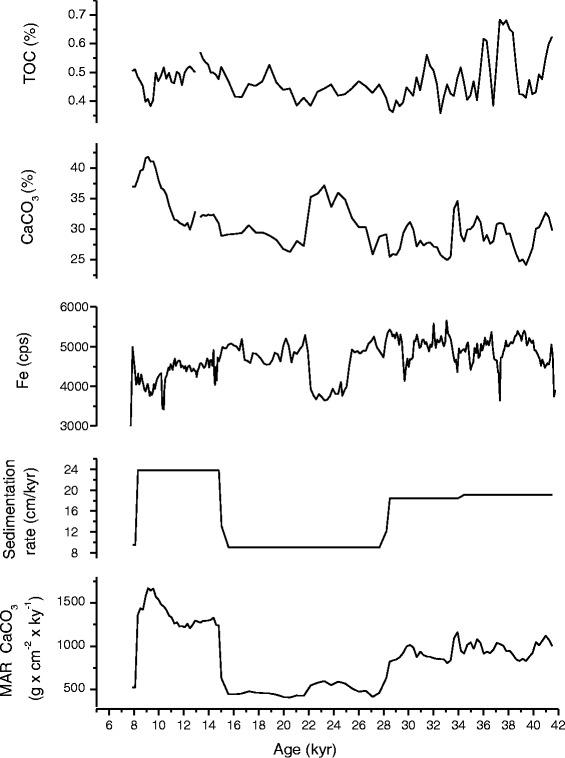
**Comparison of carbonate (CaCO**_**3**_ **) and carbonate accumulation (MAR CaCO**_**3**_ **) of the core Geob 9064 with total organic carbon (TOC) and terrigenous input (Fe).**

As previously concluded concerning the influence of sedimentation rate on TOCMAR, the carbonate accumulation rate profiles show also high dependence to sedimentation rate which makes hard to use this proxy as a proxy for paleoproductivity (Figure 5).

In addition, and as mentioned in Figure 3, the comparison between TOC and carbonate concentrations exhibits a differential variation. It was suggested that low carbonate content is probably due to dilution by terrigenous material input and/or higher organic carbon content which may cause enhanced CaCO_3_ dissolution (Emerson and Bender 1981). While the second assumption is supposed to be minor because water depth is well above Lysocline, the Figure 5 displays a parfait and synchronous negative correlation of carbonate concentration with Fe content indicating that differences in terrigenous input have a direct impact on the signal of carbonate production, we therefore deduce that variations in CaCO_3_ are due to dilution by detrital sedimentation rather than productivity changes.

#### Planktonic δ^13^C

Upwelling systems is a critical factor underlying the dependence of productivity on wind, to understand better how wind speed affect the productivity we have used the planktonic δ^13^C to predict periods of high productivity in surface waters (Berger et al. 1978).

**Figure 6:**
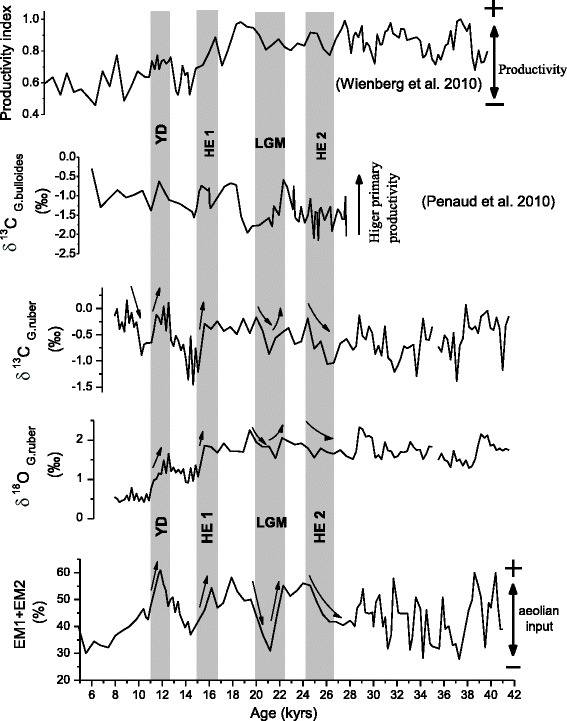
**Comparison of the productivity index estimated by Wienberg et al. (** 2010 **) and the planktonic δ** ^**13**^ **C record of the sediment core Geob 9064 (this study) with proxies related to trade wind and upwelling activity (planktonic δ** ^**18**^ **O and aeolian input).** The planktonic δ^13^C values of the core MD04-2805CQ (Penaud et al. 2010) are displayed to establish comparison with nearby location. YD: Younger Dryas; HE: Heinrich Event; LGM: Last Glacial Maximum.

Comparison of this record (Figure 6) with proxies related to trade wind and upwelling activity reveals good correlation mentioning a perfect dependence. Thus as a proxy for productivity, the carbonate fluxes and TOC content are useless in our studied area. Instead, the planktonic δ^13^C have to be considered to predict periods of high productivity.

Furthermore, a comparison with results in nearby locations reveals common and distinct patterns of paleoproductivity variations. The core Geob 9064 used in this study has been equally investigated by Wienberg et al. (2010) using foraminiferal assemblage and abundance to assess paleoproductivity conditions, her results (Figure 6) show that the Last Glacial was marked by an overall enhanced productivity and significant changes toward more oligotrophic conditions were established during the Holocene and following the end of Last glacial.

In the core MD04-2805CQ (34°30.99′N; 7°00.99′W; 859 m water depth) studied by Penaud et al. (2010), the past-primary productivity regimes were investigated on the basis of dinocyst and foraminiferal assemblage as well as on stable isotopes (O; C) and alkenones. The comparison with Wienberg et al. (2010) results indicates that the overall pattern of paleoproductivity shows comparable results except the establishment of low productive conditions exhibited during the LGM (Figure 6).

In our study, the planktonic δ^13^C values vary in a similar pattern but appear to track more perfectly the fluctuations of aeolian input and then the related upwelling activity (Figure 6), this inferring indicates that planktonic δ^13^C responds better to marine productivity changes and represents therefore a suitable proxy for paleoproductivity reconstruction in our studied area.

### Planktonic δ^13^C and paleoproductivity

#### Holocene

The planktonic δ^13^C values show a decreasing trend during the late Holocene (Figure 6), the δ^18^O records exhibits similar pattern. It′s well known that changes in planktonic δ^18^O are most likely caused by salinity fluctuations, so prominent low δ^18^O until around 10 kyr suggests increased sea surface temperature and or decreased salinity probably caused by precipitation and or riverine input. Additional evidence for interpretation of δ^13^C signal is the Aeolian input estimated by Wienberg et al. (2010) which shows low values indicating a weakening of wind strength. A combination of these results point to a general trend towards humid conditions and could promote a slow-down the upwelling system and then low productivity were reached at the late Holocene (~10 kyr).

#### Younger Dryas & Heinrich Event 1

The planktonic δ^13^C record (Figure 6) indicates two main enrichments corresponding to the Young Dryas (YD) and the Heinrich Event 1 (HE 1). With respect to Aeolian proxies, Fe content displays noticeable increasing. At the same time, the Aeolian input [Wienberg et al. (2010)] shows noteworthy increase with significant peaks indicating that wind strength reaches a maximum in these moments and highlights the activation of upwelling system. Such productive enrichment coincide with the strong shift toward high planktonic δ^18^O values which points to dryer conditions and intensified circulation i.e. strengthening of the north-eastern trade winds (Hooghiemstra et al. 1987; Marret and Turon 1994).

#### Last Glacial maximum

A prominent feature of the Last Glacial is a clear drop in productivity signal marked in the onset of the Last Glacial Maximum (LGM defined as the time interval between 19,000 to 23,000 cal-yr BP with its center at 21,000 cal-yr BP (Mix et al. 2001)) by a decreasing trend to low planktonic δ^13^C values (Figure 6), this is also supported by a clear dip in aeolian input and planktonic δ^18^O record. These observations accordingly suggest a relaxed intensity of wind strength, the establishment of weaker upwelling conditions suggesting a general convergence to evident low productivity.

#### Heinrich Event 2 (HE 2)

The onset of HE 2 (25 kyr) in core Geob 9064 is marked by a weakening of wind strength and upwelling activity marked by a decreasing trend of aeolian input and planktonic δ^18^O values (Figure 6), hence, low productivity was expressed during this interval of time.

### Organic carbon burial and deep water conditions

**Figure 7:**
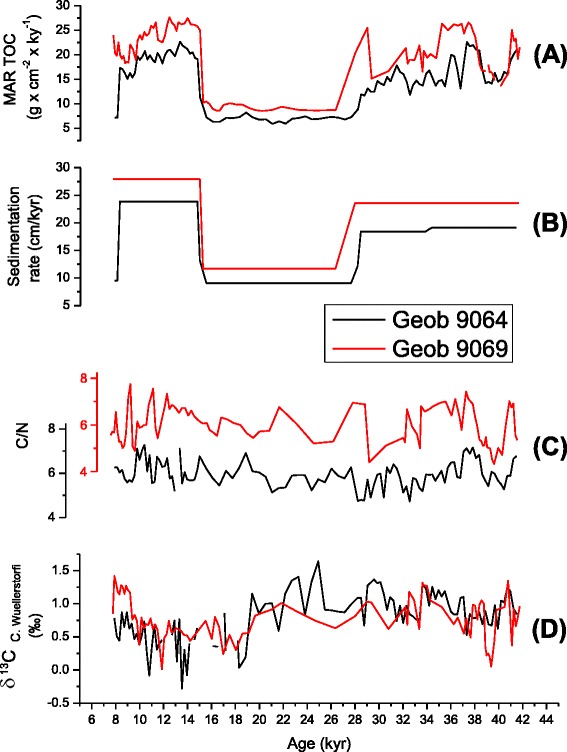
**Comparison versus age, in between (A): Total organic carbon mass accumulation rate** **(B): Sedimentation rate,** **(C): C/N ratio,** **(D): Carbon benthic isotope.**

Total organic carbon mass accumulation rate (TOC MAR) were used as a proxy for organic buried in Moroccan Atlantic Margin. This record varies in a similar manner in both cores (Figure 7A), it ranges between 5 and 40 (g.cm^−2^.ky^−1^), interglacial periods exhibit high values, while during interglacial-glacial transition we notice an abrupt decreasing to low values, this indicates that Interglacial times were marked by most pronounced organic carbon accumulation rates.

The similar patterns of C_org_ mass accumulation rates over time may be due to the geographic proximity of our cores (7 km distance) and indicate that evolution of carbon burial in our sites have subjected similar hydrological conditions. Moreover, the rapid changes of carbon burial between glacial and interglacial times reflect that the deep water production rate is essentially linked to global climate changes.

Numerous studies on the fate of organic carbon after its production in the surface water have been published (Berger et al. 1989; Stein 1991; Engel and Macko 1993; Canfield 1994; Hedges and Keil 1995). They indicate that the proportion of organic matter that escapes decomposition and becomes preserved in marine sediments can be influenced by three main factors: productivity in the surface water, sedimentary redox environment and sedimentation rate.

The reconstruction of oceanographic conditions which have contributed to a modification in the deep-water production rate during the glacial condition was assessed to understand organic carbon burial mechanism. In the following we will evaluate the changes in sedimentation rate, determine the origin of organic carbon buried and register the changes in water conditions and deep-sea circulation.

#### Sedimentation rate

The proportion of organic matter preserved depends on the total sediment accumulation rate (Heath et al. 1977; Müller and Suess 1979). High accumulation rates imply high organic carbon burial rates. In our cores, the most prominent feature of sediment accumulation rates (Figure 7B) is the wide contrast between glacial and interglacial times. The Holocene time shows highest values ranging from 23,86 cm/ky in Geob 9064 to 47,44 cm/ky in Geob 9065 and lower values during glacial time (15 – 27 kyr). The change of sedimentation patterns documents the impact of climatic change to more humid condition during the interglacial times where terrigenous supply was related to fluvial input.

Overall, high and low values of sedimentation rate are positively correlated with carbonate and C_org_ accumulation rates; this reveals that variations in sedimentation rate overwhelmingly influence the patterns of carbon burial at cores locations. Consequently, terrestial organic carbon supply may be the source of marine organic matter.

#### C/N

Sedimentary C/N ratio is widely used to distinguish between marine and terrestial organic matter. Typical terrigenous C/N ratios are > 20, whereas marine ratios range from 5 to 10 (Tyson 1995). In our study, C/N ratios (Figure 7C) between 4 and 10 indicate a general dominance of marine organic matter in the sediment. We deduce then that high sedimentation which coincides with high TOC MAR during interglacial times helps rather to preserve marine organic carbon.

#### Deep water conditions

Accumulation rate of organic carbon (TOC MAR) displayed in the (Figure 7A) marks pronounced amounts during interglacial times and it’s generally lower during glacial time, this could indicate that during interglacial we can assume conditions of sluggish deep-water circulation paralleled by an increased amount of organic matter supplied at this site, and contrary, that would suggest a more intense circulation during the glacial accompanied by an advection of small proportion of buried organic matter. The δ^13^C records of *Cibicidoides wuellerstorfi* were measured to gather information about past deep- water conditions (e.g. Curry et al. 1988; Duplessy et al. 1988; Sarnthein et al. 1994; Mackensen et al. 2001), according to Sarnthein and Tiedemann (1990) and Sarnthein et al. (1994), low values of δ ^13^C indicate low oxygenation and sluggish deep water circulation, this could account for enhanced organic matter preservation.

The comparison between the benthic δ^13^C values (Figure 7D) and the TOC MAR distribution (Figure 7A) reveals none correlation indicating that the distribution of these records varies independently; we therefore suggest the non-interference of deep-water conditions in organic carbon burial.

## Conclusion

Many proxies are currently used to reconstruct the variations of paleoproductivity, in order to determine the adequate parameter; a careful comparison was done regarding the fertilization effect of aeolian input on local upwelling activity. In addition to proxies used in previous studies, the tracing of our productivity proxies variations (TOC, carbonate and planktonic δ^13^C) indicated that the planktonic carbon isotope constitutes the best proxy that can be used to predict the paleoproductivity signal.

In this paper, the cores used showed a complete sedimentary record of the last 40 kyrs. Thus, a continuous record of paleoproductivity changes during the late glacial and interglacial times could be established. We can therefore assume that the last 40 kyrs can be divided into the following main trophic times:

- Enhanced productivity associated with intensified upwelling system during YD and HE 1.
- Weaker upwelling conditions and lower productivity are recorded during the Holocene, LGM and HE 2.

Two additional main conclusions about the organic matter that escapes decomposition and becomes preserved in marine sediments can be drawn from the present study:

1. The proportion of organic matter buried shows a clear trend to marine origin.
2. The reconstruction of oceanographic conditions which have influenced the preservation of organic matter in the sediment revealed the non-interference of deep-water conditions in organic carbon burial. On the other hand, the TOC MAR exhibits a good correlation with sedimentation rate that highlights the terrigenous influx in organic matter preservation.
